# Correlates of Aerobic Performance in Adolescents and Adults

**DOI:** 10.1002/ejsc.12311

**Published:** 2025-04-28

**Authors:** Maximiliane Thron, Ronny Kuhnert, Stefan Altmann, Friedrich Barsch, Sascha Härtel, Almut Richter, Lars Schlenker, Klaus Bös, Alexander Woll, Janis Fiedler

**Affiliations:** ^1^ Institute of Sports and Sports Science Karlsruhe Institute of Technology Karlsruhe Germany; ^2^ Robert Koch Institut Berlin Germany; ^3^ TSG ResearchLab gGmbH Zuzenhausen Germany; ^4^ Institute for Exercise and Occupational Medicine University of Freiburg Freiburg Germany; ^5^ TSG 1899 Hoffenheim Zuzenhausen Germany

**Keywords:** behavior, cardiorespiratory fitness, individual anaerobic threshold, representative, socioeconomic

## Abstract

Cardiorespiratory fitness (CRF) is considered as a main indicator of cardiovascular health and is associated with a reduced risk of noncommunicable diseases. The objective is to explore the potential behavioral, interpersonal, socioeconomic, and anthropometric factors associated with a submaximal measure for CRF among adolescents and adults in Germany. Data were drawn from a population‐based nationwide cross‐sectional study, involving 2886 male and 3034 female participants aged 14–64 who were part of the German Health Interview and Examination Survey (2008–2011) and the Examination Survey for Children and Adolescents (2014–2017). Participants completed a submaximal cycle ergometer test to determine the relative power at the individual anaerobic lactate threshold (pLT_2_) (W/kg). Multivariable survey‐weighted mixed linear regression analyses were conducted to assess the associations of potential correlates with pLT_2_. Among both sexes, being involved in physical exercise (PE) and activity (PA) and having a tertiary education was associated with hana higher pLT_2_, whereas currently smoking and classified as overweight or obese was associated with a lower pLT_2_. Among females being single and among males, a higher fruit consumption was associated with a higher pLT_2_. A lower pLT_2_ was associated with a higher waist circumference and a high junk food intake in females and being a former smoker and being single in males. Overall, PE and nonsmoking are the most important determinants for pLT_2_; therefore, policies and interventions targeting those factors would be important for preventing noncommunicable diseases. Overall, these findings can offer valuable insights for customizing prevention strategies to meet the specific needs of different subgroups.

**Summary**

- This study analyzes possible correlates of the relative power at the individual anaerobic lactate threshold (pLT_2_) as a submaximal measure for cardiorespiratory fitness (CRF) as an important predictor of cardiovascular health.
- Being engaged in physical activity and physical exercise, having a tertiary education and a higher fruit consumption, are associated with a higher pLT_2_ in adolescents and adults, whereas being a smoker, being classified as overweight or obese, having an increased waist circumference, and having a high junk‐food intake are associated with a lower pLT_2_.
- These results highlight the importance of physical activity and physical exercise as well as of a healthy lifestyle including nonsmoking, a healthy diet, and a body weight within the normal range for a higher pLT2, and therefore higher endurance performance affecting the cardiovascular health. Our findings can guide the development of targeted prevention measures for specific subgroups, which should focus on enhancing the living environment to educate and encourage individuals in making healthier life choices.

## 1 Introduction

Cardiorespiratory fitness (CRF) is considered as an important predictor of cardiovascular health and is related to a lower risk of noncommunicable diseases such as cardiovascular diseases, obesity, diabetes mellitus, or chronic respiratory diseases (Lang et al. 2024; Hurtig‐Wennlöf et al. 2007; Eisenmann et al. 2007). Therefore, CRF is an important factor to promote health by preventing the increasing prevalence of cardiovascular risk factors worldwide. Usually, CRF is assessed as maximal oxygen consumption ($\dot{V}O_{2\;\max}$) and reflects the ability of the circulatory and respiratory systems to supply oxygen to the skeletal muscles during physical exercise (Caspersen et al. 1985).

However, several concerns are raised when determining $\dot{V}O_{2\;\max}$, because maximal fitness tests to physical exhaustion are mandatory; but, for a large part of the population, maximal exhaustion is difficult to attain or even bears health risks (Meyer et al. 2005). Therefore, submaximal measures, such as lactate thresholds, as markers for aerobic endurance performance, are used to assess CRF in patients healthy subjects but also among athletes and over the entire lifespan (Faude et al. 2009). Lactate thresholds aim to define distinct processes of energy supply during exercise. The anaerobic lactate threshold reflects the highest exercise intensity that an individual can sustain for a prolonged period without substantial lactate accumulation in the blood, marking an equilibrium of lactate production and clearance and therefore of energy supply by the oxidative phosphorylation (Mader and Heck 1986). To assess the anaerobic lactate threshold (for cycling exercises), there are several definitions used in research and practice, for example, fixed anaerobic thresholds such as the 3 mmol/L threshold by Föhrenbach et al. (1987) or individual anaerobic thresholds (LT_2_) such as 1 mmol/L above the minimum lactate equivalent by Dickhuth et al. (1999). Those thresholds are commonly used as objective measures for assessing the current state of CRF and monitoring CRF during exercise interventions. Similar to $\dot{V}O_{2\;\max}$, anaerobic lactate thresholds are often normalized by body weight, as body weight relates to the amount of working muscle mass to make the data more comparable, for example, between age groups and sexes (Tuttor et al. 2018).

For the development of effective interventions aiming to reduce cardiovascular risk factors, it is crucial to understand the various factors contributing to CRF and to identify high‐risk population groups. Although it is well‐documented that fitness levels tend to decline with increasing age over the lifespan (Shephard 2009; Fiedler et al., Under Review; Tanaka and Seals 2003; Strasser and Burtscher 2018), other factors may also play significant roles in influencing CRF. In addition to genetic predispositions, physical exercise (PE) and physical activity (PA) are recognized as key elements in enhancing fitness levels. Regular engagement in PE and PA has shown to improve muscular strength and CRF, which are critical factors affecting cardiovascular health (Shephard and Bouchard 1996; Zeiher et al. 2019). Among adults, several behavioral and lifestyle factors are associated with higher levels of CRF. For instance, individuals who abstain from smoking and maintain a diet rich in healthy foods tend to exhibit better fitness levels. High occupational status, which often correlates with better access to healthcare and resources, also appears to be linked to improved CRF. Furthermore, anthropometric factors, such as maintaining a normal body mass index (BMI) and having a low waist circumference (WC), are positively related to higher CRF (i.e., relative $\dot{V}O_{2\;\max}$) (Zeiher et al. 2020). Regarding children or adolescents, some studies have indicated that maintaining a lower or normal BMI is associated with enhanced CRF (Chen et al. 2008; Peterhans et al. 2013). Additionally, lower consumption of sweetened beverages, which are often high in sugars and calories, is linked to better fitness levels (Cuenca‐García et al. 2012). The socioeconomic status of parents also plays a significant role; children from families with higher socioeconomic status tend to have better access to nutritious foods, safe environments for physical activity, and healthcare, all of which contribute to higher CRF (Lammle et al. 2012). By comprehensively understanding these factors, interventions can be tailored to address the specific needs of different population groups, thereby improving overall CRF and reducing the risk of related health issues.

However, there is a need to investigate the associations of all these factors with CRF, which were previously investigated in different studies in one single model using a representative sample and with a wider age range to get more meaningful findings for planning targeted interventions in enhancing CRF and preventing cardiovascular diseases. Therefore, we aim to determine possible correlates of the power at LT_2_ (pLT_2_) with behavioral, socioeconomic, interpersonal, anthropometric, and physical activity‐related factors among females and males living in Germany ranging from adolescents to older adults and using data from a cross‐sectional population‐based nationwide representative study.

## 2 Materials and Methods

### 2.1 Study Design

The German Health Interview and Examination Survey for Children and Adolescents (KiGGS wave 2) was conducted as a combined examination and interview survey, and the German Health Interview and Examination Survey for Adults (DEGS1) are integral components of the health monitoring program at the Robert Koch Institute in Germany. Data collection occurred between 2008 and 2011 for DEGS1 and between 2014 and 2017 for KiGGS wave 2. Participants were recruited in clusters across Germany, and the study designs, along with more detailed information, have been published elsewhere (Scheidt‐Nave et al. 2012; Mauz et al. 2017). The cycle ergometer test, coupled with blood analysis to determine lactate thresholds, was performed with a subpopulation of KiGGS wave 2 participants aged 14 years and older (including cross‐sectional and longitudinal participants; here included only until 17.9 years) and with DEGS1 participants 18–64 years of age. Additionally, questionnaires were assessed, for example, to determine behavioral factors (e.g., dietary habits, smoking, physical exercise, and activity), interpersonal (e.g., marital status), or socioeconomic factors (e.g., occupation and education). The interpersonal and socioeconomic factors of the children's parents were used for KiGGS wave 2 participants.

### 2.2 Participants

Participants in the KiGGS wave 2 and DEGS1 studies were eligible for the incremental cycle ergometer test if they passed the inspection based on the results of the Physical Activity Readiness‐Questionnaires (PAR‐Q) (J. Finger et al. 2013). Additionally, any other medical contraindications and currently elevated cardiovascular values identified during resting blood pressure measurements needed to be ruled out in consultation with a medical doctor.

The KiGGS wave 2 examination survey included a total of 9877 participants and the DEGS1 trial comprised 4947 participants. For further information regarding the inclusion criteria for the cycle ergometer test and the ergometer dataset see Fiedler et al. (Under Review).

To ensure the adequacy of the lactate curve fitting, data were screened for unrealistic outliers, and participants with fewer than 4 completed stages of the incremental cycle ergometer test were excluded. Consequently, the sample sizes were reduced to 3428 for KiGGS wave 2 and 2496 for DEGS1. To validate the results, all remaining participants' data were plotted, with lactate values on the *y*‐axis and corresponding power on the *x*‐axis. Manual screening by JF, MT, SH, and SA led to the exclusion of 101 unrealistic curves (KiGGS wave 2) and 51 unrealistic curves (DEGS1). Additionally, 19 and 12 values were adjusted for better curve fitting in the respective studies.

Finally, 34 participants from the KiGGS wave 2 dataset were removed due to their inclusion in the longitudinal and cross‐sectional study population (double entries). The final analysis included a sample of 3293 participants from KiGGS wave 2 and 2433 participants from DEGS1.

Both the KiGGS wave 2 and DEGS1 studies adhere to the guidelines outlined in the Declaration of Helsinki and the German Federal Data Protection Act. The study protocol for DEGS1 received approval from the Charité‐Universitätsmedizin Berlin ethics committee in September 2008 (Approval No. EA2/047/08) and was in accordance with the Federal and State Commissioners for Data Protection. The Ethics Commission of the Medizinische Hochschule Hannover reviewed ethical aspects and approved KiGGS wave 2 (Approval No. 2275‐2014). Participation in these studies was voluntary, and participants or their guardians received information about the study objectives, content, data protection, and provided written consent.

### 2.3 Outcome Variable: Power at Lactate Thresholds

The incremental cycle test for KiGGS wave 2 and the DEGS1 participants was conducted on a calibrated cycle ergometer with integrated blood pressure measurement and a Polar heart rate monitor (Ergosana CE 0124) (J. Finger et al. 2013; J. D. Finger et al. 2019). The participants conducted the standardized WHO protocol starting at 25 W with an incremental increase of 25 W every 2 min (Andersen et al. 1971), although they were instructed to keep a revolution rate of 60–80 rpm throughout the whole trial. Capillary blood was collected from the earlobe in the last seconds of each 2 min stage. Blood lactate concentration for each stage was analyzed utilizing Biosen C‐Line Sport (EKF‐diagnostic GmbH, Barleben, Germany). The test was stopped if participants reached an exhaustion criterion or at the end of the stage when participants reached 85% maximum heart rate (0.85 × (220−age)) for DEGS1 and participants of 18 years and older in KiGGS wave 2 and a fixed heart rate of 180 bpm for participants younger than 18 KiGGS wave 2 (J. Finger et al. 2013).

### 2.4 Potential Correlates

Based on literature research (Zeiher et al. 2019; Perumal et al. 2017; Ombrellaro et al. 2018) and theoretical frameworks (Shephard and Bouchard 1996; Zeiher et al. 2020) related to possible correlates of CRF, which were available both in the DEGS1 and KiGGS wave 2 data sets, were selected. These covariates in DEGS1 and KiGGS wave 2 were assessed using self‐administered questionnaires or measurements conducted by trained study personnel following standardized procedures (Scheidt‐Nave et al. 2012). For a more detailed description of the processing and categorization of the correlates, see Supporting Information S1.

#### 2.4.1 Behavioral Factors

Smoking status was classified as current (including occasional smoking), ex‐, or never smoking for DEGS1 and as current or never smoking for KiGGS wave 2. A food frequency questionnaire was used to assess intake frequency and portion size in the last 4 weeks for 53 food and beverage groups (Haftenberger et al. 2010). Five specific food groups were then selected ranging from health enhancing (“fruits” and “vegetables”) and health compromising products (“sugar rich drinks,” “sugar rich foods,” and “junk foods”) (Zeiher et al. 2020; Afshin et al. 2019). Categories were formed as described by Zeiher et al. (2020) for food groups and for alcohol consumption.

#### 2.4.2 Socioeconomic and Interpersonal Factors

The net equivalent income of participants' households, adjusted for their needs, was computed using data on estimated monthly net income and the number of individuals residing in the household and categorized similarly to Lampert et al. (2013). Educational level of participants (related to parents in KiGGS wave 2) was evaluated using the “Comparative Analysis of Social Mobility in Industrial Nations” (CASMIN) framework (Brauns et al. 2003). Occupational status was determined using the International Socio‐Economic Index of Occupational Status (ISEI), based on the participants' current occupation in DEGS1 and on the parents' highest occupation for KiGGS wave 2 (Ganzeboom et al. 1992). Participants' marital status for DEGS1 and of parents for KiGGS wave 2 participants was categorized based on Zeiher et al. (2020).

#### 2.4.3 Anthropometric Factors

Body weight and height were measured using portable electronic scales (SECA, Germany) and a stadiometer (Holtain, UK). The body mass index (BMI = weight [kg]/height [m]^2^) was then categorized according to the World Health Organization (WHO) guidelines (World Health Organization 2020) for the DEGS1 participants and for children and adolescents as suggested by Kromeyer‐Hauschild et al. (2015). Additionally, waist circumference (WC) was measured and categorized according to WHO guidelines for DEGS1 participants and KiGGS wave 2 participants over 18 years old (World Health Organization 2000) and for adolescents under 18 years using suggested values based on percentiles related to age and sex (Kromeyer‐Hauschild et al. 2011; Xi et al. 2019).

#### 2.4.4 Physical Activity‐Related Factors

Participants' total physical activity (PA) was assessed and categorized using the WHO recommendation (World Health Organization 2010; Krug et al. 2013). Additionally, participants were asked about how often they engage in physical exercise and also categorized based on WHO recommendations (World Health Organization 2010).

#### 2.4.5 Weighting Factors

Clustering and weighting factors were included to adjust the distribution of the sample to match those of the German population by sex, age, education, and region for all calculations (Kamtsiuris et al. 2013). The existing sample weights of the KiGGS wave 2 and DEGS1 studies were transferred to population weights, based on the population data of 14–64 year old's in Germany at the time of the KiGGS wave 2 assessment.

### 2.5 Data Processing and Statistical Analysis

The data wrangling and processing were conducted in R and RStudio (R Core Team 2022) using the tidyverse package (Wickham et al. 2019). To fit the lactate curves, the baseline data were included and a third‐degree polynomial fit was used using the lactater package (Maturana 2022). Based on the lactate curves, the performance at IAT as base lactate + 1 mmol/L was then calculated and normalized by body weight as pLT_2_ in Watt/kg (Faude et al. 2009). After processing the possible correlates as described above, the KiGGS wave 2 and DEGS1 data were merged for plotting and the statistical analysis. Descriptive results were calculated for age, pLT_2_, and the possible correlates as mean with standard deviation (SD), median with range, and number of missing data. A scatterplot was created to assess the association of pLT_2_ with age including fitted polynomial prediction lines with 95% confidence intervals (95% CIs). The svyglm function from the survey package (Lumley and Lumley 2020) was used to calculate multivariable survey‐weighted generalized linear models for complex survey data separated for sexes to analyze associations between pLT_2_ and possible correlates. Following Zeiher et al. (2020), Model 1 included age and behavioral factors without PA‐related factors, Model 2 included socioeconomic and interpersonal factors, Model 3 additionally included anthropometrics, and in Model 4, PA‐related factors were added. Additional density plots were created to show the distributions and associations of pLT_2_ with these factors across age groups (14–17, 18–24, 25–34, 35–44, 45–54, and 55–64 years) for the significant factors in the best fitting model. To assess the impact of missing data on our findings, we conducted a sensitivity analysis by multiple imputation of the missing data and the deletion of the cases where the outcome was missing (von Hippel 2007). This analysis included all participants who participated in the ergometer test (*N* = 7867). To perform the imputation, we utilized the multivariate imputation by chained equations (MICE) package (van Buuren and Groothuis‐Oudshoorn 2011). This package allows for the creation of imputation models for each variable with missing values. The algorithm then iteratively imputes multiple plausible values for the missing data, considering the uncertainty of the imputation process. This approach improves the plausibility of the condition that the missing values are missing at random. Imputation was stratified by sex and the KiGGS wave 2 and DEGS1 data were imputed separately and merged afterward. Nominal data were handled using logistic regression and categorical variables were handled using polytomous regression, whereas continuous variables were imputed with predictive mean matching. 20 datasets with 10 iterations were imputed. The imputation model incorporated all predictors, covariates, and the outcome variable.

## 3 Results

**FIGURE 1:**
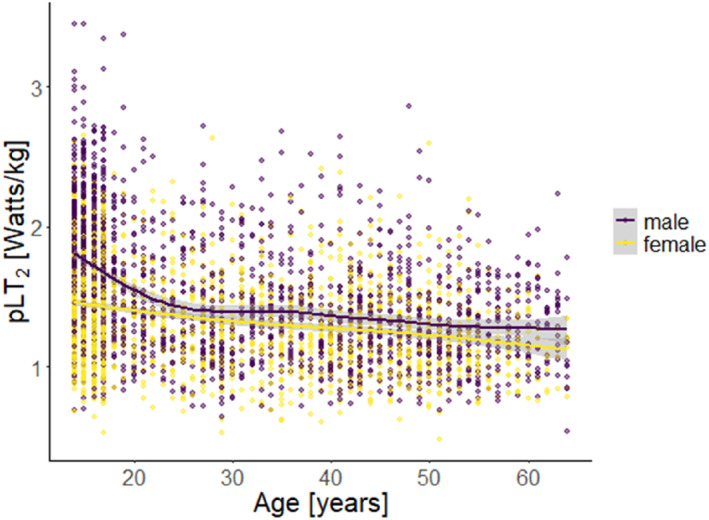
Scatterplot of the association between pLT_2_ and age. Lines represent the fitted association and gray area the 95% CI. pLT_2_, relative power at individual anaerobic threshold.

The descriptive results for male and female participants related to age, pLT_2_, and the possible correlates are shown in Table 1, and the crude and fitted association of pLT_2_ with age is shown in Figure 1.

**TABLE 1:** Descriptive results.

	Male	Female
	(*N* = 2886)	(*N* = 3034)
Age [years]		
Mean (SD)	27.8 (15.3)	28.2 (15.5)
Median [min, max]	18.0 [14.0, 64.0]	18.0 [14.0, 64.0]
pLT_2_ [W/kg]		
Mean (SD)	1.52 (0.44)	1.36 (0.34)
Median [min, max]	1.46 [0.54, 3.45]	1.32 [0.48, 2.65]
Missing	728 (25.2%)	1179 (38.9%)
Smoking status		
Never	1702 (59.0%)	1970 (64.9%)
Former	379 (13.1%)	400 (13.2%)
Current	738 (25.6%)	617 (20.3%)
Missing	67 (2.3%)	47 (1.5%)
Alcohol consumption		
Low	549 (19.0%)	581 (19.1%)
Moderate	1643 (56.9%)	1735 (57.2%)
High	544 (18.8%)	576 (19.0%)
Missing	150 (5.2%)	142 (4.7%)
Vegetables intake		
Low‐to‐moderate	1658 (57.4%)	1768 (58.3%)
High	1100 (38.1%)	1173 (38.7%)
Missing	128 (4.4%)	93 (3.1%)
Fruits intake		
Low‐to‐moderate	1671 (57.9%)	1791 (59.0%)
High	1108 (38.4%)	1185 (39.1%)
Missing	107 (3.7%)	58 (1.9%)
Junk food intake		
Low‐to‐moderate	1640 (56.8%)	1757 (57.9%)
High	1087 (37.7%)	1166 (38.4%)
Missing	159 (5.5%)	111 (3.7%)
Sugar rich drinks intake		
Low‐to‐moderate	1680 (58.2%)	1788 (58.9%)
High	1112 (38.5%)	1187 (39.1%)
Missing	94 (3.3%)	59 (1.9%)
Sugar rich foods intake		
Low‐to‐moderate	1626 (56.3%)	1742 (57.4%)
High	1076 (37.3%)	1156 (38.1%)
Missing	184 (6.4%)	136 (4.5%)
Income (% of median)		
< 60%	490 (17.0%)	518 (17.1%)
60 to < 150%	1790 (62.0%)	1921 (63.3%)
≥ 150%	601 (20.8%)	590 (19.4%)
Missing	5 (0.2%)	5 (0.2%)
Education		
Primary	509 (17.6%)	459 (15.1%)
Secondary	1564 (54.2%)	1775 (58.5%)
Tertiary	759 (26.3%)	754 (24.9%)
Missing	54 (1.9%)	46 (1.5%)
Occupational status		
Low	427 (14.8%)	377 (12.4%)
Medium	1392 (48.2%)	1690 (55.7%)
High	649 (22.5%)	596 (19.6%)
Missing	418 (14.5%)	371 (12.2%)
Marital status		
Married	1825 (63.2%)	2043 (67.3%)
Single	698 (24.2%)	579 (19.1%)
Separated/divorced/widowed	301 (10.4%)	364 (12.0%)
Missing	62 (2.1%)	48 (1.6%)
Body mass index		
Normal weight	1632 (56.5%)	2085 (68.7%)
Underweight	135 (4.7%)	143 (4.7%)
Overweight	770 (26.7%)	528 (17.4%)
Obese	347 (12.0%)	276 (9.1%)
Missing	2 (0.1%)	2 (0.1%)
Waist circumference		
Normal	1861 (64.5%)	1736 (57.2%)
Increased	505 (17.5%)	643 (21.2%)
Strongly increased	513 (17.8%)	647 (21.3%)
Missing	7 (0.2%)	8 (0.3%)
Physical exercise per week		
No physical exercise	644 (22.3%)	805 (26.5%)
0–2 h	765 (26.5%)	987 (32.5%)
Over 2 h	1377 (47.7%)	1180 (38.9%)
Missing	100 (3.5%)	62 (2.0%)
Physical activity		
Guidelines not met	2166 (75.1%)	2613 (86.1%)
Guidelines met	637 (22.1%)	361 (11.9%)
Missing	83 (2.9%)	60 (2.0%)

Abbreviations: pLT_2_, relative individual anaerobic threshold and SD, standard deviation.

The weighted multivariable generalized linear regression analysis shows that the model, including age (linear for males and cubic for females), behavioral, socioeconomic and interpersonal factors, and anthropometrics as well as PA‐related factors, has the best fit for both sexes to assess associations with the pLT_2_ (see Table 2). The first three models are presented in Supporting Information S1 (Tables S1–S3).

**TABLE 2:** Final model for the associations between pLT_2_ and correlates in male and female participants.

Predictors	Final model male (*N* = 1598)			Final model female (*N* = 1425)		
	Estimates	95% CI	*p*	Estimates	95% CI	*p*
Intercept	1.46	1.32–1.60	**< 0.001**	1.26	1.17–1.36	**<** **0.001**
Age						
Age	0.00	−0.01 to −0.00	**0.002**	−1.83	−3.08 to −0.57	**0.004**
Age (cubic)				−0.64	−1.68 to 0.40	0.229
Smoking status						
Never	Ref.					
Former	−0.08	−0.15 to −0.02	**0.012**	−0.04	−0.09 to 0.01	0.157
Current	−0.12	−0.17 to −0.07	**<** **0.001**	−0.05	−0.10 to −0.01	**0.023**
Alcohol consumption						
Low	Ref.					
Moderate	0.02	−0.04 to 0.08	0.509	−0.01	−0.06 to 0.04	0.636
High	0.03	−0.05 to 0.10	0.462	0.02	−0.04 to 0.08	0.595
Vegetable intake						
Low‐to‐moderate	Ref.					
High	0.02	−0.03 to 0.06	0.447	0.02	−0.02 to 0.06	0.312
Fruit intake						
Low‐to‐moderate	Ref.					
High	0.08	0.03–0.13	**0.002**	0.03	−0.01 to 0.07	0.117
Junk food intake						
Low‐to‐moderate	Ref.					
High	−0.01	−0.05 to 0.04	0.822	−0.04	−0.07 to −0.01	**0.015**
Sugar rich drinks intake						
Low‐to‐moderate	Ref.					
High	−0.01	−0.06 to 0.03	0.560	0.00	−0.03 to 0.04	0.953
Sugar rich foods intake						
Low‐to‐moderate	Ref.					
High	0.01	−0.03 to 0.05	0.659	−0.01	−0.05 to 0.02	0.523
Income (% of median)						
< 60%	Ref.					
60 to < 150%	−0.01	−0.06 to 0.05	0.839	0.05	−0.01 to 0.10	0.093
≥ 150%	0.00	−0.07 to 0.07	0.963	0.05	−0.01 to 0.11	0.11
Education						
Primary	Ref.					
Secondary	0.05	−0.00 to 0.11	0.056	0.00	−0.05 to 0.05	0.934
Tertiary	0.11	0.04–0.19	**0.003**	0.07	0.00–0.14	**0.037**
Occupational status						
Low	Ref.					
Medium	0.00	−0.06 to 0.05	0.902	−0.01	−0.07 to 0.04	0.602
High	0.00	−0.08 to 0.08	0.970	−0.01	−0.07 to 0.06	0.794
Marital status						
Married, living together	Ref.					
Single	−0.06	−0.12 to −0.00	**0.048**	0.08	0.04–0.12	**<** **0.001**
Separated/divorced/widowed	0.05	−0.05 to 0.14	0.332	0.00	−0.06 to 0.07	0.909
Body mass index						
Normal weight	Ref.					
Underweight	0.14	0.02–0.26	**0.022**	0.10	0.01–0.19	**0.036**
Overweight	−0.13	−0.19 to −0.08	**<** **0.001**	−0.11	−0.16 to −0.06	**<** **0.001**
Obese	−0.28	−0.38 to −0.19	**<** **0.001**	−0.23	−0.29 to −0.16	**<** **0.001**
Waist circumference						
Normal	Ref.					
Increased	−0.04	−0.10 to 0.02	0.156	−0.12	−0.16 to −0.07	**<** **0.001**
Strongly increased	−0.06	−0.14 to 0.02	0.121	−0.16	−0.22 to −0.10	**<** **0.001**
Physical exercise per week						
No physical exercise	Ref.					
0–2 h	0.10	0.05–0.15	**<** **0.001**	0.10	0.06–0.14	**<** **0.001**
> 2 h	0.25	0.18–0.31	**<** **0.001**	0.21	0.15–0.27	**<** **0.001**
Physical activity						
WHO guidelines not met	Ref.					
WHO guidelines met	0.12	0.06–0.18	**<** **0.001**	0.05	0.00–0.10	**0.043**
*R* ^2^	**0.346**			**0.431**		

Abbreviations: CI, confidence interval; *p*, significance value (bold – *p* < 0.05); *R* ^2^, coefficient of determination; and WHO, World Health Organization.

The final model shows that among males, the pLT_2_ decreases over the lifespan, that is, from 17 to 64 years (*β* = −0.00; CI = −0.01 to −0.00; *p* = 0.002). Although cubic age has shown to be the best fit among females, it does not have a significant influence on the pLT_2_ (*β* = −0.64; CI = −1.68 to 0.40; *p* = 0.229; and Table 2).

Being a current smoker is associated with a lower pLT_2_ among males and females (*β* = −0.12; CI = −0.15 to −0.02; and *p* < 0.001 and *β* = −0.05; CI = −0.10 to −0.01; and *p* = 0.023, respectively), whereas being a former smoker is only associated with a lower pLT_2_ in males compared to being a nonsmoker (*β* = 0.08; CI = −0.15 to −0.02; and *p* = 0.012). Males with a high fruit intake show a higher pLT2 (*β* = 0.08; CI = 0.03–0.13; and *p* = 0.002) compared to those with low‐to‐moderate fruit intake. Although this not being evident in females, they show a lower pLT_2_ when having a high compared to a low‐to‐moderate junk food intake (*β* = −0.08; CI = −0.07 to −0.01; and *p* = 0.015).

In addition, males and females with a tertiary education (of boys' and girls' parents for KiGGS wave 2 participants) have a higher pLT_2_ compared to those with a primary education (*β* = 0.11; CI = 0.04–0.19; and *p* = 0.003 and *β* = 0.07; CI = 0.00–0.14; and *p* = 0.037, respectively). Related to marital status (of boys' and girls' parents for KiGGS wave 2 participants), males' pLT_2_ decreases by *β* = −0.06 W/kg (CI = −0.12 to −0.00 and *p* = 0.048), whereas females' pLT_2_ increases by *β* = 0.08 W/kg (CI = 0.04–0.12 and *p* < 0.001) when being single in comparison to being married and living together.

In addition, an inverse association was observed between BMI and the pLT_2_ with males and females having a higher pLT2 being underweight (*β* = 0.14; CI = 0.02–0.26 and; *p* = 0.022 and *β* = 0.10; CI = 0.01–0.19; and *p* = 0.036, respectively) but lower pLT_2_ being overweight (*β* = −0.13; CI = −0.19 to −0.08; and *p* < 0.001 and −*β* = 0.11; CI = −0.16 to −0.06; and *p* < 0.001, respectively) and obese (*β* = −0.28; CI = −0.38 to −0.19; and *p* < 0.001 and *β* = −0.23; CI = −0.29 to −0.16; and *p* < 0.001, respectively) compared to being classified as normal weight. Similarly, females with an increased and strongly increased WC show a lower pLT_2_ compared to those with a normal WC (*β* = −0.12; CI = −0.16 to −0.07; and *p* < 0.001 and *β* = −0.16; CI = −0.22 to −0.10; and *p* < 0.001, respectively); however, these results were not evident in males.

Among males and females, the pLT_2_ increases with increasing physical weekly exercise: up to 2 h of PE (*β* = 0.10; CI = 0.05–0.15; and *p* < 0.001 and *β* = 0.10; CI = 0.06–0.14; and *p* < 0.001, respectively) and more than 2 h of PE per week (*β* = 0.25; CI = 0.18–0.31; and *p* < 0.001 and *β* = 0.21; CI = 0.15–0.27; and *p* < 0.001, respectively) in comparison to no PE. Similarly, males and females who met the WHO recommendations for PA showed higher pLT_2_ than those who did not meet the recommendations (*β* = 0.12; CI = 0.06–0.18; and *p* < 0.001 and *β* = 0.05; CI = 0.00–0.10; and *p* = 0.043, respectively).

The sensitivity analysis using imputated data shows similar results (see Table S4). However, in the imputed model, the pLT_2_ does not show a significant increase when males had a tertiary education and no decrease when being single, whereas females show an increasing pLT_2_ with a secondary education and an intermediate income.

## 4 Discussion

This study aimed to determine possible correlates of CRF operationalized by pLT_2_ with behavioral, socioeconomic, interpersonal, anthropometric, and PA‐related factors among females and males living in Germany ranging from adolescents to older adults using representative data from two population‐based nation‐wide studies. In summary, females currently smoking, with a high junk food intake, classified as overweight or obese and a (strongly) increased WC show a lower pLT_2_, whereas females with a tertiary education, being single, and being involved in PE and PA show a higher pLT_2_. Among males, being a former or current smoker, single, overweight, or obese was associated with a lower pLT_2_, whereas a higher fruit consumption, tertiary education, and being engaged in PE and PA was associated with a higher pLT_2_.

### 4.1 Age Differences

A decreasing CRF with increasing age has been confirmed in previous studies (Fiedler et al. Under Review; Zeiher et al. 2020). However, age per se is not a cause for a decreasing CRF, whereas different mechanisms related to the aging process can have an impact on CRF. Related to this, a decline in physical fitness over the lifespan is well‐described by a reduction in cardiac output, mitochondrial function, or muscle mass atrophy (Weiss et al. 2006; Short and Nair 2001).

### 4.2 Behavioral Factors

Most studies investigating the effects of smoking on CRF with a cross‐sectional design found contrary results with no, positive, or negative associations between smoking and CRF (Zeiher et al. 2019; Thai et al. 2014). However, the negative effects of smoking on health and especially the respiratory system with an increasing risk of respiratory and cardiovascular diseases are well‐documented (Freund et al. 1993). Alongside several longitudinal studies (Bernaards et al. 2003; Jackson et al. 2009; Fleg et al. 2005), those findings support the results of negative effects of being a former or current smoker on pLT2 in our study. It is noteworthy to mention that age might influence the association between smoking and the pLT2 in our study, as the option former smokers was not assessed in the KiGGS wave 2 data due to the age of KiGGS wave 2 participants. Additionally, when interpreting the association between smoking and the pLT2 or CRF, a meaningful effect on health and performance, reported by a change of 5% (Poole et al. 2021; Pettee Gabriel et al. 2023), should be taken into account. This is represented in male and not in female participants of our study.

Studies investigating the association of the quality of diets with CRF where high‐quality food is linked to vegetables and fruits and showing higher CRF, whereas low‐quality food, linked to high‐fat dairy products, meats, and sweets, is associated with lower CRF (Eslami et al. 2020). These findings might be attributed to healthier lifestyle choices in general of, for example, having a healthier diet with less energy‐dense foods while exercising regularly (Romieu et al. 2017). In our study, we only included a smaller selection of foods for the specific food groups whereby the rating of the quality of the consumed food is limited. That could be a reason, why we only observed an association of fruit intake with the pLT_2_ among males, which is in line with previous studies showing higher CRF for men with higher fruit intake (Zeiher et al. 2020; Shikany et al. 2013; Howe et al. 2016). Among females, the fruit intake did not show positive associations with pLT_2_; however, the sensitivity analysis using imputed data showed that also females with a higher fruit intake have a higher pLT_2_. Although, the low *β* (below 5%) indicates a nonrelevant association of fruit intake on the pLT_2_ in females (Poole et al. 2021; Pettee Gabriel et al. 2023). Additionally, a higher junk food intake was associated with a lower pLT_2_ among females but also with a nonrelevant association (*β* below 5%). Additionally, vegetable intake, sugar rich foods, and drinks consumption were not associated with the pLT_2_. Finally, even though alcohol is considered a strong neurotoxin and the consumption is not recommended from a health perspective, no association between alcohol consumption and pLT_2_ was found in this study.

### 4.3 Socioeconomic and Interpersonal Factors

The final model showed that income was not associated with pLT_2_ among boys' parents/men, but sensitivity analysis indicated that girls' parents/women with an intermediate compared to a low net equivalent household income have a higher pLT_2_. Alongside the findings that tertiary education is positively related to the pLT_2_, those results reflect the outcomes of previous studies showing that children of families with a higher socioeconomic status (Lammle et al. 2012; Jin and Jones‐Smith 2015) and adults with a higher income and education have a higher CRF (Ombrellaro et al. 2018). Although this is not supported by the occupational status in our study, it is assumed that subjects with a higher education on the one hand have a higher income, which can improve accessibility of health‐related infrastructure, and on the other hand are informed about the health benefits of PA and PE, which leads to a higher CRF (Ombrellaro et al. 2018). Those findings suggest to enhance health‐related interventions among families with a lower socioeconomic status to reach children at an early age and support them and their families to achieve a health‐oriented behavior over the life span (Lammle et al. 2012).

Interestingly, being single has shown to be positively associated with the pLT_2_ among females but negatively associated with the pLT_2_ among males compared to those who are married (marital status of boys' and girls' parents for KiGGS wave 2 participants). However, related to the males, the low *β* indicates a nonrelevant association and the effect disappears in the sensitivity analysis has to be treated with caution. Overall, studies on this topic are scarce. Contrary to our results, Ortega et al. (2011) found that men who were single had a higher CRF compared to those who were married but no associations were evident among women. In line with our research, being divorced or widowed was not associated with the CRF neither in men nor in women (Ortega et al. 2011). Single women might have more free time or experience social pressure, hence being more active or being younger than those who are already married, divorced, separated, or widowed, which might include an age, effect on pLT_2_. However, it remains unclear why men's results are ambiguous or why girls with a single parent are fitter than those with married parents and boys are not.

### 4.4 Anthropometric Factors

BMI as well as WC were both strongly associated with pLT_2_. Underweight males and females showed higher values, whereas overweight or obese males and females had lower values of pLT_2_ compared to normal weight, which is in line with the current literature (Zeiher et al. 2020; Chen et al. 2008; Carnethon et al. 2005). However, the few studies investigating associations of underweight with CRF showed contrasting results with a higher CRF for underweight women but not men (Zeiher et al. 2020) or with a lower CRF in underweight men but not women (Hung et al. 2014). The contrast to our results might be due to our data including also adolescents and not only adults and the subgroup of underweight participants being very small in our data. Additionally, late‐developing adolescents might be underweight and therefore could distort those results.

In line with the results regarding BMI, an increased or strongly increased WC was associated with a lower pLT_2_ among females compared to a normal WC; however, males' data only showed a tendency. Those results are supported by recent studies investigating associations of CRF with abdominal obesity measured by WC in adults and adolescents (Zeiher et al. 2019, 2020; Dagan et al. 2013; Buchan et al. 2014). Research indicates that higher CRF is linked to lower levels of abdominal fat and visceral adipose tissue. Therefore, it can be assumed that greater health benefits of CRF may be due to the reduced abdominal fat in individuals with higher fitness levels (Wong et al. 2004). Additionally, a study examining $\dot{V}O_{2\;\max}$ in relation to fat‐free mass also found a negative relationship with obesity in men and women (Duvigneaud et al. 2008). It is noteworthy to mention that the significant association of the pLT_2_ with BMI and WC might be also due to the definition of the pLT_2_ being relative to body weight, which is also reflected in the BMI and WC.

### 4.5 PA‐Related Factors

**FIGURE 2:**
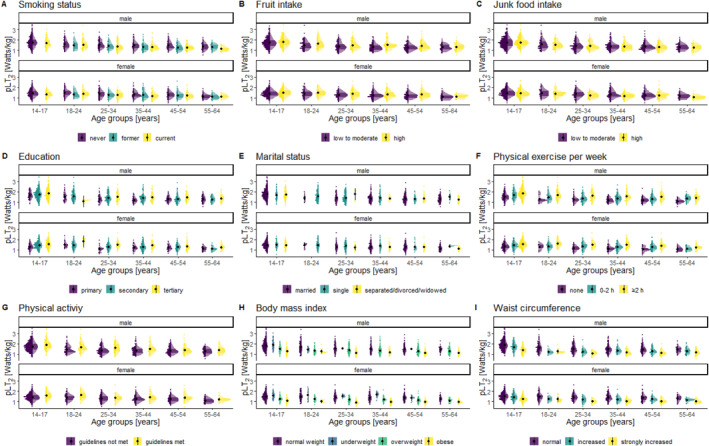
Density plots of the pLT_2_ related to age groups and the significant correlates for males and females. pLT_2_, relative power at individual anaerobic threshold.

We observed strong associations of PE and PA with pLT_2_ and the highest associations were found for more than 2 h of PE per week. It is well‐established through empirical evidence that most individuals experience both short‐term and long‐term physiological adaptations in response to regular physical exercise and training, leading to improved CRF. Generally, higher amounts and intensities of activity result in greater CRF improvements (Lin et al. 2015). Our findings support this dose–response relationship, showing further increases in CRF with higher amounts of weekly PE, which is consistent in every age group ranging from the age 14 to 64 (see Figure 2F,G).

### 4.6 Strengths and Limitations

Our study bears the major strength of using population‐based weighted and hence representative data, allowing for more general interpretations of associations between behavioral, interpersonal, socioeconomic, and anthropometric factors and CRF. Additionally, the outcome variable pLT_2_ was assessed using a highly standardized and well‐established procedures ensuring high‐quality data (J. Finger et al. 2013). However, it is noteworthy to mention that the cross‐sectional design does not allow for conclusions regarding causality between the correlates and CRF. Since the sampling periods were in 2008–2011 and 2014–2017, rapidly transforming societies (e.g., the increasing use of electronical cigarettes or increasing obesity over the last years) could influence the direct transfer of our findings to present and future societies. Another limitation of our study is that we could not include some potential correlates due to difference between the DEGS1 and KiGGS wave 2 survey, for example, social support. In addition, due to the survey‐based design, most of the variables are based on self‐reporting by the participants. Hence, bias cannot be ruled out. Despite numerous efforts to enhance participation, account for unequal sampling probabilities, and align the sample distribution with official population statistics, the relatively low response rate may have introduced selection bias. Even with the use of weighting factors, certain population groups, such as individuals with less interest in health behavior, may still be underrepresented in our study. Additionally, the PAR‐Q and thus excluding participants due to the use of medications or cardiovascular conditions as prerequisites of the incremental cycling test may have resulted in a healthier population profile potentially distorting the findings.

## 5 Conclusion

We conclude that multiple factors across behavioral, interpersonal, and socioeconomic as well as anthropometric domains are linked to CRF in the general adolescent and adult population in Germany. Additionally, the pLT_2_ served as a submaximal measure for CRF and can be implemented in future studies allowing to include a wider spectrum of participants. Our findings can guide the development of targeted prevention measures for specific subgroups, for example, with a primary education. For example, the link between pLT_2_ and the consumption of specific foods suggests that various healthy behaviors should be considered collectively in addition to isolated analyses. Additionally, enhancing PA and PE should be addressed and supported over the life span. Other aspects of a healthy lifestyle, such as a balanced diet and not smoking, are also important; excess weight or abdominal fat mass should be avoided. Effective measures should address not only individual behaviors but also include environmental and policy level interventions. Therefore, efforts to promote these behaviors should focus on enhancing the living environment to facilitate healthier choices.

## Ethics Statement

Both the KiGGS wave 2 and DEGS1 studies adhere to the guidelines outlined in the Declaration of Helsinki and the German Federal Data Protection Act. The study protocol for DEGS1 received approval from the Charité‐Universitätsmedizin Berlin ethics committee in September 2008 (Approval No. EA2/047/08) and was in accordance with the Federal and State Commissioners for Data Protection. The Ethics Commission of the Medizinische Hochschule Hannover reviewed ethical aspects and approved KiGGS wave 2 (Approval No. 2275‐2014). Participation in these studies was voluntary, and participants or their guardians received information about the study objectives, content, data protection, and provided written consent.

## Conflicts of Interest

The authors declare no conflicts of interest.

## Supporting information

Supporting Information S1

## Data Availability

The datasets used for the analyses of the current study could be available upon request.
