# Supplementary material for: Correlates of Aerobic Performance in Adolescents and Adults
Source: Eur J Sport Sci. 2025 Apr 28;25(5):e12311. doi: 10.1002/ejsc.12311 (PMC12036346; doi:10.1002/ejsc.12311)
Supplement: Supplementary file 1 — Supporting Information S1 [file EJSC-25-e12311-s001.docx]

**Potential Correlates**

Based on literature research [16, 27, 28] and theoretical frameworks [15, 17] related to possible correlates of CRF which were available both in the DEGS1 and KiGGS wave 2 data sets, were selected. These covariates in DEGS1 and KiGGS wave 2 were assessed using self-administered questionnaires or measurements conducted by trained study personnel following standardized procedures [22].

*Behavioral Factors*

Smoking status was classified as current (including occasional smoking), ex- or never smoking for DEGS1 and as current or never smoking for KiGGS wave 2. A food frequency questionnaire was used to assess intake frequency and portion size in the last four weeks for 53 food and beverage groups [29]. Five specific food groups were then selected ranging from health enhancing (“fruits” and “vegetables”) and health compromising products (“sugar rich drinks”, “sugar rich foods” and “junk foods”) [17, 30]. As described in detail by Zeiher et al. [17], the intake quantities of food-groups were calculated by combining the frequency of intake and the portion size of the relevant food and beverage groups. Based on the quantities for each food group, two categories were formed using sex-specific quintiles: low to moderate intake (quintiles 1–3) and high intake (quintiles 4–5). For beer, wine, spirits, and cocktails, ethanol in grams per day was calculated by multiplying the calculated quantity of each alcoholic beverage with standard ethanol content and then added as one food group. Three categories using sex-specific quintiles were defined as low alcohol consumption (quintile 1), medium alcohol consumption (quintiles 2–4), and high alcohol consumption (quintile 5) [17].

*Socioeconomic and Interpersonal Factors*

The net equivalent income of participants’ households, adjusted for their needs, was computed using data on estimated monthly net income and the number of individuals residing in the household. The income was categorized into three groups: below 60%, 60–150%, and above 150% of the median net household equivalent income. These categories represent income levels below the relative poverty line, intermediate income, and relatively high income, respectively [31]. Educational level was evaluated using the ‘Comparative Analysis of Social Mobility in Industrial Nations’ (CASMIN) framework, which classified participants (related to parents in KiGGS wave 2) into three categories: primary, secondary, and tertiary education [32]. Occupational status was determined using the International Socio-Economic Index of Occupational Status (ISEI), based on the participants’ current occupation in DEGS1 and on the parents’ highest occupation for KiGGS wave 2. The variable was further divided into three groups: low occupational status (quintile 1), medium occupational status (quintiles 2–4), and high occupational status (quintile 5) [17]. Participants’ marital status was categorized as single, married (while living together), and separated/divorced/widowed for DEGS1 and of parents for KiGGS wave 2 participants.

*Anthropometric Factors*

Body weight and height were measured using portable electronic scales (SECA, Germany) and a stadiometer (Holtain, UK). The body mass index (BMI = weight [kg] / height [m]^2^) was then categorized according to the World Health Organization (WHO) guidelines [34] for the DEGS1 participants: Underweight (BMI < 18.5), normal weight (18.5 ≤ BMI < 25), overweight (25 ≤ BMI < 30), and obese (BMI ≥ 30). For KiGGS wave 2 participants, BMI was classified using percentiles as suggested for children and adolescents [35]: Underweight (< percentile 10), normal weight (percentile 10 until < 90), overweight (percentile 90 until < 97), and obese (> percentile 97). Additionally, waist circumference (WC) was measured at the smallest site between the lowest rib and the superior border of the iliac crest using a flexible, non-stretchable measurement tape. WC was further categorized as ‘normal,’ ‘increased,’ or ‘strongly increased’ according to WHO guidelines for DEGS1 participants and KiGGS wave 2 participants over 18 years old [36] and for adolescents under 18 years using suggested values based on percentiles related to age and sex [37, 38].

*Physical Activity-related Factors*

To assess total physical activity (PA), participants were asked about the number of days in an average week when they engaged in physical activities intense enough to cause sweating or breathlessness. If participants reported any PA, they were further queried about the duration of PA on those days. Participants were categorized into two groups using the WHO recommendation [39, 40]: <2.5 h per week (guidelines not met) and ≥2.5 h per week (guidelines met) for DEGS1 participants; and <60 min per day (guidelines not met) and ≥60 min per day (guidelines met) for KiGGS wave 2 participants. Additionally, participants were asked about how often they engage in physical exercise (PE; as a subset of physical activity that is planned, structured, and repetitive, with the specific goal of improving or maintaining physical fitness) and also categorized based on WHO recommendations [39]: no PE, >0 h – <2 h of PE per week, ≥2 h per week for both DEGS1 and KiGGS wave 2 participants.

Supplementary Table 1: Model 1 with behavioral data (excluding PA and PE) for males and females

|  | **Final Model male**  **(N=1874)** | | | **Final Model female**  **(N=1631)** | | |
| --- | --- | --- | --- | --- | --- | --- |
| *Predictors* | *Estimates* | *95% CI* | *p* | *Estimates* | *95% CI* | *p* |
| **Intercept** | 1.71 | 1.61 – 1.81 | **<0.001** | 1.32 | 1.25 – 1.38 | **<0.001** |
| **Age** |  |  |  |  |  |  |
| Age | -0.01 | -0.01 – -0.01 | **<0.001** | -5.96 | -7.59 – -4.33 | **<0.001** |
| Age (cubic) |  |  |  | -0.45 | -1.47 – 0.58 | 0.391 |
| **Smoking status** | |  |  |  |  |  |
| Never | ref. | | | | | |
| Former | -0.14 | -0.21 – -0.07 | **<0.001** | -0.04 | -0.10 – 0.01 | 0.139 |
| Current | -0.14 | -0.19 – -0.09 | **<0.001** | -0.11 | -0.16 – -0.07 | **<0.001** |
| **Alcohol consumption** | |  |  |  |  |  |
| Low | ref. | | | | | |
| Moderate | 0.03 | -0.03 – 0.09 | 0.350 | 0.07 | 0.01 – 0.13 | **0.019** |
| High | 0.01 | -0.08 – 0.09 | 0.897 | 0.15 | 0.09 – 0.22 | **<0.001** |
| **Vegetable intake** | |  |  |  |  |  |
| Low to moderate | ref. | | | | | |
| High | 0.03 | -0.02 – 0.08 | 0.230 | 0.06 | 0.01 – 0.11 | **0.027** |
| **Fruit intake** | |  |  |  |  |  |
| Low to moderate | ref. | | | | | |
| High | 0.11 | 0.06 – 0.16 | **<0.001** | 0.06 | 0.01 – 0.10 | **0.015** |
| **Junk food intake** | |  |  |  |  |  |
| Low to moderate | ref. | | | | | |
| High | -0.02 | -0.07 – 0.04 | 0.538 | -0.09 | -0.13 – -0.04 | **<0.001** |
| **Sugar rich drinks intake** | |  |  |  |  |  |
| Low to moderate | ref. | | | | | |
| High | -0.01 | -0.06 – 0.03 | 0.553 | 0.00 | -0.04 – 0.04 | 0.983 |
| **Sugar rich foods intake** | |  |  |  |  |  |
| Low to moderate | ref. | | | | | |
| High | 0.00 | -0.04 – 0.05 | 0.859 | 0.01 | -0.03 – 0.06 | 0.515 |
| **R^2^** | **0.135** | | | **0.147** | | |

CI – Confidence interval; p – significance value (bold – p<0.05); R^2^ – Coefficient of determination.

Supplementary Table 2: Model 2 with behavioral (excluding PA and PE) and socioeconomic data for males and females

|  | **Final Model male**  **(N=1634)** | | | **Final Model female**  **(N=1445)** | | |
| --- | --- | --- | --- | --- | --- | --- |
| *Predictors* | *Estimates* | *95% CI* | *p* | *Estimates* | *95% CI* | *p* |
| **Intercept** | 1.58 | 1.43 – 1.73 | **<0.001** | 1.17 | 1.06 – 1.28 | **<0.001** |
| **Age** |  |  |  |  |  |  |
| Age | -0.01 | -0.01 – -0.00 | **<0.001** | -4.19 | -5.69 – -2.70 | **<0.001** |
| Age (cubic) |  |  |  | -1.36 | -2.57 – -0.16 | **0.027** |
| **Smoking status** | |  |  |  |  |  |
| Never | ref. | | | | | |
| Former | -0.14 | -0.21 – -0.07 | **<0.001** | -0.06 | -0.12 – 0.00 | 0.066 |
| Current | -0.15 | -0.21 – -0.09 | **<0.001** | -0.08 | -0.13 – -0.03 | **0.003** |
| **Alcohol consumption** | |  |  |  |  |  |
| Low | ref. | | | | | |
| Moderate | 0.02 | -0.04 – 0.08 | 0.503 | 0.04 | -0.03 – 0.10 | 0.249 |
| High | 0.01 | -0.08 – 0.09 | 0.848 | 0.08 | 0.01 – 0.15 | **0.021** |
| **Vegetable intake** | |  |  |  |  |  |
| Low to moderate | ref. | | | | | |
| High | 0.01 | -0.03 – 0.06 | 0.559 | 0.04 | -0.01 – 0.09 | 0.143 |
| **Fruit intake** | |  |  |  |  |  |
| Low to moderate | ref. | | | | | |
| High | 0.10 | 0.05 – 0.16 | **<0.001** | 0.06 | 0.01 – 0.10 | **0.012** |
| **Junk food intake** | |  |  |  |  |  |
| Low to moderate | ref. | | | | | |
| High | -0.01 | -0.06 – 0.05 | 0.816 | -0.08 | -0.12 – -0.04 | **<0.001** |
| **Sugar rich drinks intake** | |  |  |  |  |  |
| Low to moderate | ref. | | | | | |
| High | -0.01 | -0.06 – 0.04 | 0.716 | 0.00 | -0.04 – 0.05 | 0.904 |
| **Sugar rich foods intake** | |  |  |  |  |  |
| Low to moderate | ref. | | | | | |
| High | 0.01 | -0.03 – 0.06 | 0.554 | -0.00 | -0.04 – 0.04 | 0.972 |
| **Income (% of median)** | |  |  |  |  |  |
| <60% | ref. | | | | | |
| 60 to <150% | -0.01 | -0.08 – 0.06 | 0.826 | 0.07 | 0.00 – 0.14 | **0.049** |
| ≥150% | 0.02 | -0.06 – 0.10 | 0.654 | 0.11 | 0.03 – 0.18 | **0.008** |
| **Education** |  |  |  |  |  |  |
| Primary | ref. | | | | | |
| Secondary | 0.07 | 0.00 – 0.13 | **0.037** | 0.04 | -0.03 – 0.10 | 0.277 |
| Tertiary | 0.15 | 0.07 – 0.24 | **0.001** | 0.12 | 0.03 – 0.20 | **0.006** |
| **Occupational status** | |  |  |  |  |  |
| Low | ref. | | | | | |
| Medium | 0.02 | -0.04 – 0.08 | 0.475 | 0.01 | -0.05 – 0.08 | 0.672 |
| High | 0.02 | -0.08 – 0.12 | 0.664 | 0.07 | -0.01 – 0.14 | 0.096 |
| **Marital status** | |  |  |  |  |  |
| Married, living together | ref. | | | | | |
| Single | 0.01 | -0.06 – 0.07 | 0.857 | 0.11 | 0.06 – 0.17 | **<0.001** |
| Separated/divorced/ widowed | 0.10 | 0.00 – 0.20 | **0.050** | -0.01 | -0.08 – 0.07 | 0.886 |
| **R^2^** | **0.149** | | | **0.192** | | |

CI – Confidence interval; p – significance value (bold – p<0.05); R^2^ – Coefficient of determination.

Supplementary Table 3: Model 3 with behavioral (excluding PA and PE), socioeconomic, and anthropometric data for males and females

|  | **Final Model male**  **(N=1633)** | | | **Final Model female**  **(N=1443)** | | |
| --- | --- | --- | --- | --- | --- | --- |
| *Predictors* | *Estimates* | *95% CI* | *p* | *Estimates* | *95% CI* | *p* |
| **Intercept** | 1.61 | 1.48 – 1.74 | **<0.001** | 1.34 | 1.25 – 1.43 | **<0.001** |
| **Age** |  |  |  |  |  |  |
| Age | -0.00 | -0.01 – -0.00 | **<0.001** | -2.61 | -3.88 – -1.33 | **<0.001** |
| Age (cubic) |  |  |  | -0.80 | -1.89 – 0.30 | 0.152 |
| **Smoking status** | |  |  |  |  |  |
| Never | ref. | | | | | |
| Former | -0.11 | -0.17 – -0.04 | **0.001** | -0.03 | -0.09 – 0.02 | 0.245 |
| Current | -0.13 | -0.18 – -0.08 | **<0.001** | -0.06 | -0.11 – -0.02 | **0.005** |
| **Alcohol consumption** | |  |  |  |  |  |
| Low | ref. | | | | | |
| Moderate | 0.02 | -0.04 – 0.07 | 0.582 | 0.01 | -0.05 – 0.07 | 0.693 |
| High | 0.01 | -0.07 – 0.09 | 0.826 | 0.05 | -0.02 – 0.12 | 0.129 |
| **Vegetable intake** | |  |  |  |  |  |
| Low to moderate | ref. | | | | | |
| High | 0.03 | -0.02 – 0.07 | 0.273 | 0.04 | -0.01 – 0.08 | 0.084 |
| **Fruit intake** | |  |  |  |  |  |
| Low to moderate | ref. | | | | | |
| High | 0.10 | 0.05 – 0.15 | **<0.001** | 0.05 | 0.01 – 0.09 | **0.017** |
| **Junk food intake** | |  |  |  |  |  |
| Low to moderate | ref. | | | | | |
| High | -0.00 | -0.05 – 0.05 | 0.909 | -0.06 | -0.10 – -0.03 | **<0.001** |
| **Sugar rich drinks intake** | |  |  |  |  |  |
| Low to moderate | ref. | | | | | |
| High | -0.00 | -0.05 – 0.04 | 0.917 | 0.01 | -0.03 – 0.04 | 0.751 |
| **Sugar rich foods intake** | |  |  |  |  |  |
| Low to moderate | ref. | | | | | |
| High | 0.01 | -0.03 – 0.06 | 0.624 | -0.00 | -0.04 – 0.04 | 0.970 |
| **Income (% of median)** | |  |  |  |  |  |
| <60% | ref. | | | | | |
| 60 to <150% | 0.00 | -0.06 – 0.06 | 0.993 | 0.05 | 0.00 – 0.11 | **0.049** |
| ≥150% | 0.02 | -0.06 – 0.09 | 0.655 | 0.05 | -0.01 – 0.12 | 0.115 |
| **Education** |  |  |  |  |  |  |
| Primary | ref. | | | | | |
| Secondary | 0.07 | 0.01 – 0.12 | **0.014** | -0.01 | -0.06 – 0.05 | 0.788 |
| Tertiary | 0.13 | 0.05 – 0.21 | **0.001** | 0.06 | -0.01 – 0.14 | 0.097 |
| **Occupational status** | |  |  |  |  |  |
| Low | ref. | | | | | |
| Medium | 0.00 | -0.05 – 0.06 | 0.935 | 0.01 | -0.05 – 0.07 | 0.697 |
| High | 0.00 | -0.08 – 0.09 | 0.925 | 0.05 | -0.03 – 0.13 | 0.213 |
| **Marital status** | |  |  |  |  |  |
| Married, living together | ref. | | | | | |
| Single | -0.03 | -0.09 – 0.03 | 0.383 | 0.09 | 0.04 – 0.13 | **<0.001** |
| Separated/divorced/ widowed | 0.07 | -0.02 – 0.16 | 0.130 | -0.00 | -0.08 – 0.07 | 0.900 |
| **Body mass index** |  |  |  |  |  |  |
| Normal weight | ref. | | | | | |
| Underweight | 0.11 | -0.00 – 0.22 | 0.051 | 0.07 | -0.03 – 0.16 | 0.173 |
| Overweight | -0.13 | -0.19 – -0.07 | **<0.001** | -0.10 | -0.16 – -0.05 | **<0.001** |
| Obese | -0.24 | -0.33 – -0.14 | **<0.001** | -0.24 | -0.32 – -0.17 | **<0.001** |
| **Waist circumference** |  |  |  |  |  |  |
| Normal | ref. | | | | | |
| Increased | -0.07 | -0.13 – -0.01 | **0.032** | -0.12 | -0.17 – -0.07 | **<0.001** |
| Strongly increased | -0.13 | -0.21 – -0.06 | **0.001** | -0.16 | -0.23 – -0.10 | **<0.001** |
| **R^2^** | **0.250** | | | **0.367** | | |

CI – Confidence interval; p – significance value (bold – p<0.05); R^2^ – Coefficient of determination.

Supplementary Table 4: Final model for males and females with imputed data

|  | **Final Model male**  **(N=2156)** | | | **Final Model female**  **(N=1853)** | | |
| --- | --- | --- | --- | --- | --- | --- |
| *Predictors* | *Estimates* | *95% CI* | *p* | *Estimates* | *95% CI* | *p* |
| **Intercept** | 1.45 | 1.33 – 1.57 | **0.000** | 1.20 | 1.12 – 1.30 | **<0.001** |
| **Age** |  |  |  |  |  |  |
| Age | -0.00 | -0.01 – -0.00 | **0.002** |  |  |  |
| Age (cubic) |  |  |  | -0.46 | -1.19 – 0.27 | 0.219 |
| **Smoking status** | |  |  |  |  |  |
| Never | ref. | | | | | |
| Former | -0.08 | -0.13 – -0.01 | **0.015** | -0.04 | -0.09 – 0.00 | 0.057 |
| Current | -0.08 | -0.13 – -0.03 | **0.001** | -0.07 | -0.11 – -0.03 | **0.001** |
| **Alcohol consumption** | |  |  |  |  |  |
| Low | ref. | | | | | |
| Moderate | 0.03 | -0.02 – 0.08 | 0.212 | 0.01 | -0.03 – 0.06 | 0.588 |
| High | 0.03 | -0.03 – 0.09 | 0.376 | 0.06 | -0.00 – 0.12 | 0.054 |
| **Vegetable intake** | |  |  |  |  |  |
| Low to moderate | ref. | | | | | |
| High | 0.02 | -0.03 – 0.06 | 0.484 | 0.01 | -0.03 – 0.05 | 0.659 |
| **Fruit intake** | |  |  |  |  |  |
| Low to moderate | ref. | | | | | |
| High | 0.08 | 0.04 – 0.12 | **<0.001** | 0.04 | -0.00 – 0.07 | **0.031** |
| **Junk food intake** | |  |  |  |  |  |
| Low to moderate | ref. | | | | | |
| High | -0.02 | -0.06 – 0.03 | 0.460 | -0.05 | -0.02 – -0.02 | **0.002** |
| **Sugar rich drinks intake** | |  |  |  |  |  |
| Low to moderate | ref. | | | | | |
| High | 0.00 | -0.04 – 0.04 | 0.937 | 0.02 | -0.02 – 0.05 | 0.304 |
| **Sugar rich foods intake** | |  |  |  |  |  |
| Low to moderate | ref. | | | | | |
| High | 0.01 | -0.03 – 0.05 | 0.631 | 0.01 | -0.02 – 0.05 | 0.383 |
| **Income (% of median)** | |  |  |  |  |  |
| <60% | ref. | | | | | |
| 60 to <150% | -0.01 | -0.06 – 0.04 | 0.767 | 0.05 | 0.00 – 0.09 | **0.045** |
| ≥150% | 0.00 | -0.06 – 0.04 | 0.978 | 0.03 | -0.02 – 0.09 | 0.261 |
| **Education** |  |  |  |  |  |  |
| Primary | ref. | | | | | |
| Secondary | 0.04 | -0.00 – 0.09 | 0.106 | 0.01 | -0.04 – 0.05 | 0.797 |
| Tertiary | 0.07 | 0.00 – 0.14 | 0.061 | 0.07 | 0.01 – 0.14 | **0.019** |
| **Occupational status** | |  |  |  |  |  |
| Low | ref. | | | | | |
| Medium | 0.00 | -0.06 – 0.05 | 0.893 | 0.02 | -0.04 – 0.07 | 0.564 |
| High | 0.01 | -0.07 – 0.09 | 0.863 | 0.03 | -0.03 – 0.09 | 0.344 |
| **Marital status** | |  |  |  |  |  |
| Married, living together | ref. | | | | | |
| Single | -0.05 | -0.10 – -0.01 | 0.088 | 0.08 | 0.04 – 0.12 | **<0.001** |
| Separated/divorced/ widowed | 0.03 | -0.05 – 0.11 | 0.500 | 0.01 | -0.05 – 0.08 | 0.695 |
| **Body mass index** |  |  |  |  |  |  |
| Normal weight | ref. | | | | | |
| Underweight | 0.01 | -0.11 – 0.13 | 0.907 | 0.17 | 0.06 – 0.28 | **0.004** |
| Overweight | -0.16 | -0.20 – -0.10 | **<0.001** | -0.12 | -0.18 – -0.07 | **<0.001** |
| Obese | -0.30 | -0.39 – -0.21 | **<0.001** | -0.22 | -0.29 – -0.16 | **<0.001** |
| **Waist circumference** |  |  |  |  |  |  |
| Normal | ref. | | | | | |
| Increased | -0.04 | -0.09 – 0.01 | 0.147 | -0.10 | -0.15 – -0.06 | **<0.001** |
| Strongly increased | -0.07 | -0.14 – 0.01 | 0.147 | -0.14 | -0.21 – -0.08 | **<0.001** |
| **Physical exercise per week** |  |  |  |  |  |  |
| No physical exercise | ref. | | | | | |
| 0 to 2 h | 0.11 | 0.07 – 0.16 | **<0.001** | 0.09 | 0.06 – 0.13 | **<0.001** |
| > 2 h | 0.24 | 0.18 – 0.30 | **<0.001** | 0.21 | 0.15 – 0.26 | **<0.001** |
| **Physical activity** |  |  |  |  |  |  |
| WHO guidelines not met | ref. | | | | | |
| WHO guidelines met | 0.11 | 0.06 – 0.17 | **<0.001** | 0.06 | 0.01 – 0.11 | **0.011** |
| **R^2^** | **0.346** | | | **0.431** | | |

CI – Confidence interval; p – significance value (bold – p<0.05); R^2^ – Coefficient of determination.
